# Correlation analysis between tongue hyperspectral imaging and tongue coating microbiota and feasibility study for early screening of type 2 diabetes mellitus status

**DOI:** 10.3389/fendo.2026.1936566

**Published:** 2026-09-18

**Authors:** Zhao-wei Liu, Yan Zhang, Lei Fu, Jing-ya Xie, Xin-yu Zhang, Ming Liu, Jing Zhao

**Affiliations:** 1 Tianjin University of Traditional Chinese Medicine, Tianjin, China; 2 Binhai New Area Hospital of TCM, Tianjin, China; 3 Institute of Biomedical Engineering, Chinese Academy of Medical Sciences and Peking Union Medical College, Tianjin, China

**Keywords:** disease risk prediction, hyperspectral tongue imaging, machine learning algorithms, tongue coating microbiota, type 2 diabetes mellitus

## Abstract

**Objective:**

To investigate the feasibility of applying hyperspectral imaging technology combined with machine learning algorithms for early non-invasive screening of type 2 diabetes mellitus (T2DM).

**Methods:**

In this study, tongue images of 153 subjects were acquired using a hyperspectral imaging system. Concurrently, 53 tongue coating samples were collected for 16S rRNA sequencing. Spearman correlation analysis was performed between tongue hyperspectral data and tongue coating microbiota. Finally, machine learning was integrated to construct a T2DM disease status identification model based on tongue hyperspectral data.

**Results:**

The experimental results revealed that 10 genera exhibited significant differences among the three groups (*P* < 0.05). Among these, the relative abundance of *unclassified_p_Bacillota*, *Pseudoleptotrichia*, *Xylanibacter*, *Gemella*, *norank_f_Mitochondria*, and *Enhydrobacter* showed significant correlations with hyperspectral tongue images (*P* < 0.05). The T2DM disease status identification models constructed based on tongue hyperspectral data achieved favorable modeling results, with the optimal model demonstrating ACC = 0.8966 and AUC = 0.8029.

**Conclusion:**

The results of this study indicate that certain correlations exist between tongue hyperspectral features and tongue coating microbial genera in T2DM patients at different disease stages. The T2DM disease status identification model constructed based on tongue hyperspectral imaging and machine learning algorithms can be used for the identification of HC, IGR, and T2DM, and this method may provide a noninvasive and rapid screening approach for early identification of T2DM.

## 1 Introduction

Diabetes mellitus (DM) is a chronic metabolic disease with high global prevalence, among which type 2 diabetes mellitus (T2DM) accounts for more than 90% of cases, with a higher incidence in males than in females. Epidemiological data indicate (1) that by 2024, the global prevalence of diabetes among adults has reached 11.11%, and it is projected to increase to 13% by 2050. Furthermore, the proportion of individuals with impaired glucose regulation (IGR) is as high as 12%, imposing a heavy burden on society and families. Currently, the diagnosis of T2DM relies on venous blood glucose or glycosylated hemoglobin A1c (HbA1c), which presents limitations including invasiveness, poor patient compliance, and high costs. Therefore, exploring noninvasive and convenient methods for early identification of T2DM is crucial for delaying disease progression.

Alterations in T2DM disease status influence changes in the oral microenvironment. Studies have shown (2) that *Corynebacterium* is significantly reduced in the tongue coating of individuals with prediabetes, whereas *Johnsonella* is significantly increased. Additional research has demonstrated (3) that *Streptococcus*, *Rothia*, *Prevotella*, *Neisseria*, and *Porphyromonas* constitute the primary structure of the salivary microbiota in T2DM patients. Further analysis revealed that in T2DM patients with disease duration exceeding 10 years, *Streptococcus*, *Weissella*, *unclassified_g_Streptococcus*, *Prevotella_jejuni*, and *Weissella_cibaria* were significantly enriched; whereas in T2DM patients with disease duration less than 10 years, *Capnocytophaga*, *Treponema refringens*, and *Capnocytophaga granulosa* were significantly enriched. Imbalance of the oral microbiota not only induces oral diseases such as dental caries and periodontal disease but also affects intestinal microecological balance through the oral-gut axis. Following destruction of the intestinal barrier, inflammatory factors and endotoxins enter the bloodstream, thereby exacerbating systemic pathological conditions including cardiovascular disease, digestive system disorders, and metabolic diseases (4).

As the initial part of the digestive tract, the tongue is closely associated with the oral microbiome. The tongue is composed of muscular tissue with abundant blood supply (5, 6). Changes in tongue color are intimately related to tissue blood flow status, and alterations in tongue morphology and texture can result from dietary factors, dehydration, anemia, fungal colonization, vitamin levels, activation of the NF-κB signaling pathway, and microvascular circulatory disturbances in the tongue (7). Compared with the fingertips and arms, tongue color can more accurately reflect the internal microcirculatory status of the human body (8). Tongue coating is a biofilm attached to the dorsal surface of the tongue, composed of shed epithelial cells, saliva, blood cells, food residues, and oral microbial communities (9). Changes in its structure and composition can reflect alterations in the local microbial ecology and underlying systemic pathological conditions. From an optical perspective, variations in tongue tissue reflectance are determined by the combined effects of multiple biological tissue properties. The wavelength absorption differences between hemoglobin and deoxyhemoglobin provide information on local oxygenation and blood perfusion status. Meanwhile, tissue water content, mucosal thickness, keratinization, collagen composition, and extracellular matrix organization influence light scattering behavior within tissues (10). Furthermore, biofilms formed by tongue coating microbiota can modify the surface microstructure of the tongue coating, thereby affecting optical scattering characteristics. Under disease conditions, inflammation, microbial dysbiosis, and metabolic disturbances may collectively induce alterations in the spectral reflectance patterns of tongue tissues (11). Therefore, the tongue image can convey substantial pathological information, and its clinical value has been validated in the diagnosis and treatment of various chronic and metabolic diseases (12, 13). Disease status identification based on the tongue image offers advantages including simple operation, safety, high efficiency, noninvasiveness, absence of pain, lack of side effects, and real-time monitoring, and has gained widespread international recognition.

In T2DM, distinct tongue coating microbial profiles have been associated with metabolic phenotypes and tongue coating characteristics. Wang et al. reported that T2DM patients with yellow tongue coating exhibited enrichment of *Lactobacillus* in both tongue coating and gut microbiota, accompanied by alterations in insulin, glucagon, body mass index (BMI), and homeostatic model assessment of β-cell function, suggesting that tongue coating features may reflect coordinated changes within the oral-gut microbial ecosystem and host glucose metabolism (14). Deng, J. et al. (15) integrated tongue image features with oral and gut microbiomes across healthy controls (HC), IGR, and T2DM patients, revealing progressive alterations in both tongue features and microbial composition. They found that the abundance of *Firmicutes* along the oral-gut axis was associated with white greasy tongue coating, while machine-learning model integrating tongue image parameters with *Escherichia* and *Porphyromonas-A* significantly improved the discrimination between IGR and T2DM. Consistently, a multi-omics study (2) integrating tongue coating 16S rRNA sequencing, gut metagenomics, and plasma metabolomics identified altered abundances of *Corynebacterium* and *Johnsonella* in IGR and demonstrated coordinated microbial shifts associated with carbohydrate and lipid metabolism, further supporting the potential of tongue coating microbiota as a marker of systemic metabolic changes. Beyond diabetes, similar microbiome–tongue associations have been observed in other metabolic disorders. For example, in metabolic dysfunction-associated steatotic liver disease, combining tongue image features, clinical parameters, and microbial markers such as *Streptococcus* and *Rothia* significantly improved non-invasive diagnostic performance (16). Recent studies (17) demonstrated that machine-learning models integrating microbiome signatures and tongue characteristics could distinguish different metabolic disease phenotypes. These findings establish a framework linking metabolic disorders, tongue coating microbiota, and quantitative tongue imaging. However, most previous studies have relied on conventional RGB-based tongue imaging, extracting color, texture, and coating-related features, or have primarily integrated microbiome data and tongue images at the diagnostic model level. Direct characterization of the wavelength-specific relationship between tongue microbiota and tissue optical properties remains limited. Hyperspectral imaging, which simultaneously captures spatial and continuous spectral information associated with hemoglobin absorption, tissue water content, microcirculation, and surface scattering, provides an opportunity to extend microbiome-tongue imaging research from macroscopic phenotypic associations toward quantitative optical phenotyping.

Hyperspectral imaging technology (18) integrates multiple disciplines including spectroscopy, imaging science, and computer science, characterized by rich spectral information and the fusion of spectral and spatial data. Based on the theory of light reflection, it can simultaneously acquire spatial and spectral information of the tongue within the range of 400~1000 nm at nanometer-level spectral resolution. Different bands reflect different physical quantities and information of the measured object: the visible light band primarily reflects color characteristics, while the near-infrared band is associated with physiological and biochemical indicators such as water content, microcirculatory status, and tissue oxygenation levels. It can be employed to detect and analyze the composition and structure of substances, revealing subtle changes in tongue mucosal microvasculature, hemoglobin, and advanced glycation end products (AGEs), thereby providing a novel objective approach for early disease diagnosis. In recent years, with the advancement of computer data processing capabilities, progress in image recognition technology, and continuous improvement of machine learning algorithms, hyperspectral imaging technology has been widely applied in the novel diagnosis of common diseases including peripheral vascular disease (19), cancer (20), retinal disease (21), diabetic foot (22), and dermatosis (23). In the field of tongue image research, Liang, A. et al. (24) first combined hyperspectral tongue imaging with machine learning for the identification of menstrual disorders, validating the feasibility of hyperspectral technology in disease diagnosis.

Based on the above analysis, this study aims to explore the association between lingual hyperspectral features and the microbial community structure of tongue coating in T2DM, and to investigate the feasibility of using lingual hyperspectral imaging combined with machine learning algorithms for identifying T2DM disease status. Tongue coating samples and lingual hyperspectral data were collected from T2DM, IGR, and HC subjects. The microbial community structure of tongue coating was analyzed using 16S rRNA sequencing technology to screen for characteristic flora associated with T2DM, and differences in lingual hyperspectral features among the three groups were analyzed. Subsequently, the association between lingual hyperspectral features and the microbial community structure of tongue coating in T2DM was explored. Finally, machine learning models, including support vector machine (SVM), random forest (RF), and partial least squares discriminant analysis (PLS-DA), were constructed based on hyperspectral tongue features to evaluate their capability for T2DM identification, thereby establishing a non-invasive, objective, and efficient early screening technique for diabetes and providing theoretical basis and technical support for clinical early screening of diabetes. The experimental workflow is illustrated in **Figure 1**.

**Figure 1:**
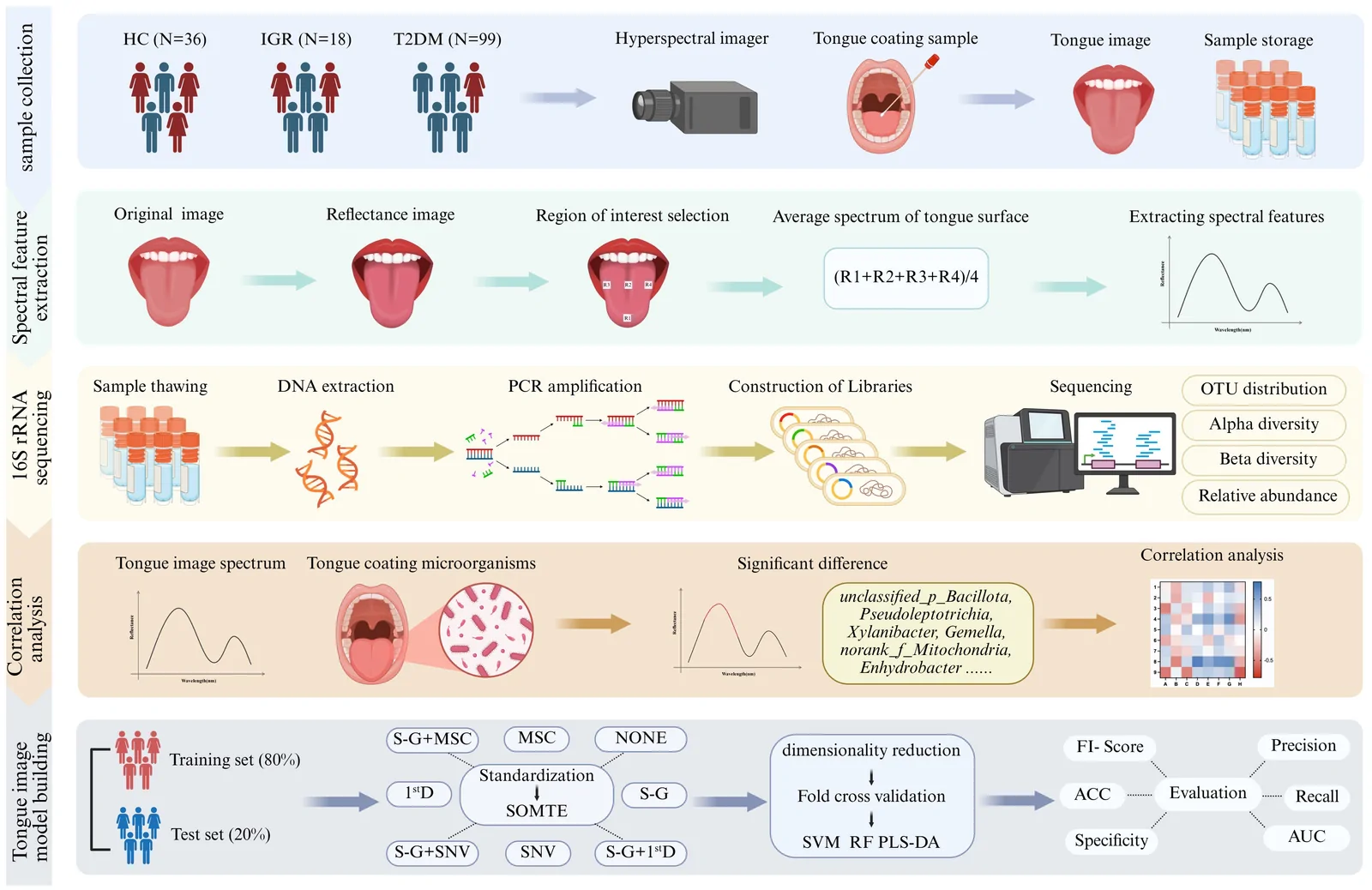
Experimental workflow.

## 2 Methods

### 2.1 Research object

This study was approved by the Ethics Committee of Tianjin University of Traditional Chinese Medicine (TJUTCM-EC20190004). All procedures involving human participants were conducted in accordance with the ethical standards of the institutional and national research committee and with the 1964 Helsinki Declaration and its later amendments. All participants voluntarily enrolled in the study after providing fully informed consent and signed written informed consent forms. Between February 2025 and August 2025, a total of 153 hyperspectral tongue images were collected at the Fourth Affiliated Hospital of Tianjin University of Traditional Chinese Medicine, including 99 participants with T2DM, 18 with IGR, and 36 healthy controls (HC). All participants underwent fasting and/or postprandial blood glucose testing (via venous blood or fingertip blood sampling), and their clinical baseline data were comprehensively documented. Concurrently, tongue coating samples were collected from 53 individuals who had already undergone hyperspectral tongue imaging, comprising 18 HC, 10 IGR, and 25 T2DM patients.

### 2.2 Diagnostic criteria

#### 2.2.1 Diagnostic criteria for T2DM

According to the 2025 edition of the *Standards of Care in Diabetes* published by the American Diabetes Association (ADA) (25), a diagnosis of type 2 diabetes can be confirmed in individuals presenting with classic symptoms of diabetes (polydipsia, polyuria, polyphagia, and unexplained weight loss) who also meet any one of the following criteria:

1. Random venous plasma glucose ≥ 11.1 mmol/L;
2. Fasting venous plasma glucose ≥ 7.0 mmol/L;
3. 2 h plasma glucose during OGTT ≥ 11.1 mmol/L;
4. HbA1c ≥ 6.5%.
5. For individuals without typical symptoms, a repeat test on a subsequent day is required to confirm the diagnosis.

#### 2.2.2 Diagnostic criteria for IGR

According to the 2025 edition of the *Standards of Care in Diabetes* published by the American Diabetes Association (ADA) (25), prediabetes is defined as a state of elevated blood glucose levels that do not meet the diagnostic criteria for diabetes, encompassing impaired fasting glucose (IFG) and impaired glucose tolerance (IGT).

1. IFG:5.6 mmol/L ≤ FPG < 6.9 mmol/L;
2. IGT:7.8 mmol/L ≤ 2 h plasma glucose during OGTT < 11.0 mmol/L;
3. HbA1c:5.7%∼6.4%.

### 2.3 Inclusion and exclusion criteria

#### 2.3.1 Inclusion criteria

1. Meeting the aforementioned diagnostic criteria for diabetes or prediabetes;
2. Aged between 25 and 80 years;
3. Able to cooperate and complete tongue image acquisition as required;
4. Complete clinical data available and written informed consent obtained.

#### 2.3.2 Exclusion criteria for tongue coating microbiome collection

1. Use of antibiotics, immunosuppressants, hormonal medications, or high-dose probiotics within the past 6 months;
2. History of gastrointestinal surgery or coexisting digestive system diseases such as cholangitis, cirrhosis, or pancreatitis;
3. Presence of oral mucosal diseases, oral abscesses, oral cancer, fungal infections, or other oral conditions affecting the entire mouth;
4. Receipt of dental or oral treatment within the past 6 months;
5. Systemic diseases other than T2DM, such as cerebral infarction, heart disease, nephropathy, or malignancies;
6. Infectious diseases such as hepatitis B, AIDS, or syphilis;
7. Pregnant or lactating women;
8. Psychiatric disorders, poor compliance, inability to cooperate, or failure to complete the experiment as required.

#### 2.3.3 Exclusion criteria for tongue image acquisition

1. Patients with type 1 diabetes or gestational diabetes;
2. Patients with other severe primary diseases, such as cerebrovascular accident, myocardial infarction, congestive heart failure, or malignant tumors;
3. Patients with acute or severe diabetic complications, including diabetic ketoacidosis, hyperosmolar hyperglycemic state, severe hypoglycemia, or acute infection complicated with diabetes;
4. Patients with chronic heavy alcohol consumption, viral hepatitis, thyroid dysfunction, or other related disorders;
5. Patients with poor compliance or inability to cooperate, those unable to complete tongue image acquisition as required, or those with tongue coating stained by medications or food;
6. Patients with psychiatric disorders, severe intellectual or cognitive impairment, altered consciousness, nasogastric feeding, mechanical ventilation, or other conditions resulting in unclear verbal expression, inability to accurately describe subjective symptoms, or failure to complete tongue protrusion for imaging.

Note: Patients meeting any of the above criteria were excluded from the study.

#### 2.3.4 Exclusion criteria

1. Individuals with incomplete or unclear tongue images;
2. Those who were erroneously enrolled despite not meeting the inclusion criteria.

### 2.4 Sample collection

#### 2.4.1 Tongue coating sample collection

Participants were instructed to abstain from eating, drinking, tooth brushing, and tongue scraping for 12 h prior to sampling. Before sample collection, participants rinsed their mouths twice with sterile water, and their oral condition was inspected. Tongue coating samples were collected from the posterior-middle to anterior-middle region of the dorsal tongue surface and immediately immersed in phosphate-buffered saline. The swab sponge was pressed against the tube wall repeatedly for 20 seconds to ensure thorough transfer of microorganisms into the buffer solution. Cryovials were labeled with identification numbers and collection dates, placed in racks, and transferred into ice boxes packed with ice packs to create a low-temperature environment that inhibited microbial growth. All samples were transferred to a -80 °C freezer within 2 h. Upon completion of sample collection, all specimens were shipped to the laboratory for sequencing analysis.

#### 2.4.2 Hyperspectral tongue image information acquisition

The hyperspectral tongue imaging system consisted of a hyperspectral imager (HY-6010-S, Hangzhou Gaopu Imaging Technology Co., Ltd.), symmetrically positioned halogen light sources (Philips, 50 W), and a computer system equipped with image acquisition software (Lenovo-PC, Intel^®^ Core™ i5–7400 CPU @ 3.00 GHz, 4 GB RAM). The spectral range was set from 380 to 1030 nm, encompassing 300 continuous bands, with an integration time of 311ms and a spatial resolution of 400 × 397 pixels.

To minimize the influence of ambient light, a fully enclosed imaging chamber was employed to isolate external light interference, thereby ensuring uniform and stable illumination conditions and guaranteeing high-quality, highly reproducible image acquisition. Prior to tongue image acquisition, the operator connected the power supply and inspected the light sources and imaging parameters; the instrument was preheated for 30 minutes to achieve a stable illumination environment. A reference image of a white diffuse reflectance standard with 98% reflectance was acquired. The outer edges and contact surfaces of the instrument were disinfected with 75% ethanol to prevent cross-infection. Participants were instructed to rinse their mouths three times with purified water to remove oral debris, and were provided with tongue protrusion training. Each participant was seated with the body slightly leaning forward, positioned their face within the mask of the tongue imaging device with full contact against the edges, and was instructed to extend the tongue naturally out of the mouth with the tongue body relaxed, the dorsal surface flattened, and the tip pointing downward to fully expose the tongue body. This posture was maintained for 2–3 seconds, and 2–3 images were captured per participant to ensure image clarity and adequate tongue exposure. Upon completion of acquisition, clear and complete images were selected and organized according to the assigned information codes. The entire acquisition procedure was performed by the same operator under identical environmental conditions.

### 2.5 Data processing

#### 2.5.1 DNA extraction, library construction and sequencing

The frozen samples stored at -80 °C were thawed and thoroughly homogenized. A 1.5 mL aliquot of bacterial culture broth was aspirated, centrifuged for 10 minutes, and the supernatant was discarded to obtain the sediment. Total genomic DNA of the tongue coating microbial community was extracted using the E.Z.N.A.^®^ Soil DNA Kit (Omega Bio-tek, Norcross, GA, USA) according to the manufacturer’s instructions. The extracted DNA was assessed for quality by 1% agarose gel electrophoresis, and the concentration and purity were measured using a NanoDrop 2000 (Thermo Scientific, USA). Upon passing quality control, the DNA was subjected to PCR amplification. The V3-V4 hypervariable region of the 16S rRNA gene was amplified using the barcoded forward primer 338F (5’-ACTCCTACGGGAGGCAGCAG-3’) and the reverse primer 806R (5’-GGACTACHVGGGTWTCTAAT-3’). The PCR products were purified and subsequently evaluated by 1.5% agarose gel electrophoresis. Qualified products were used to construct sequencing libraries following the standard protocol of the NEXTFLEX^®^ Rapid DNA-Seq Kit. Finally, paired-end sequencing of the amplicon libraries was performed on the Illumina PE300/PE250 platform (Illumina, San Diego, CA, USA).

#### 2.5.2 Extraction of spectral features

To minimize interference from non-uniform system illumination and camera dark current noise during the experiment, uniformity correction was performed on the raw images to ensure the accuracy and reliability of subsequent spectral data analysis. Hyperspectral images were imported using HHIT hyperspectral image processing software, and system reflectance calibration was conducted with a white diffuse reflectance standard of 98% reflectance as the correction reference.

In this study, a standardized region of interest (ROI) extraction method based on tongue anatomical regions was adopted. Considering the variations in tongue size, shape, and imaging position among individuals, a proportional division method (1/5 segmentation) was first adopted to minimize the influence of spatial variation on spectral feature extraction. Specifically, the anterior 1/5 regions of the tongue were defined as the tongue tip region, the bilateral 1/5 regions were defined as the left and right tongue edge regions, and the posterior 1/5 region was considered the tongue root region. The central region of the tongue was defined as the tongue middle region (**Figure 2B**). However, the tongue root region was not included in ROI extraction because its spectral signals are more susceptible to variations in imaging angle and individual anatomical differences during hyperspectral acquisition, resulting in relatively lower spectral stability and insufficient inter-subject comparability. Subsequently, a 30 × 30 pixels ROI was selected within each predefined tongue region. ROI selection was not based on fixed pixel coordinates but was manually adjusted according to the actual tongue morphology and relative anatomical position of each subject to ensure spatial correspondence among subjects. Specifically, the tongue tip ROI was selected from the distal region along the midline of the tongue; the tongue middle ROI was positioned at the central point of the tongue midline; and the left and right tongue edge ROIs were selected bilaterally at the same horizontal level as the tongue middle ROI, thereby maximizing consistency of the extracted regions across subjects. Finally, the mean reflectance spectrum within each ROI was calculated, and spectral information ranging from 380 to 1030 nm was extracted as the original spectrum (OS) feature. Furthermore, the reflectance spectra from the four regions, including the tongue tip, tongue middle, left tongue edge, and right tongue edge, were combined to construct a tongue surface reflectance dataset for subsequent statistical analysis and machine learning model development.

**Figure 2:**
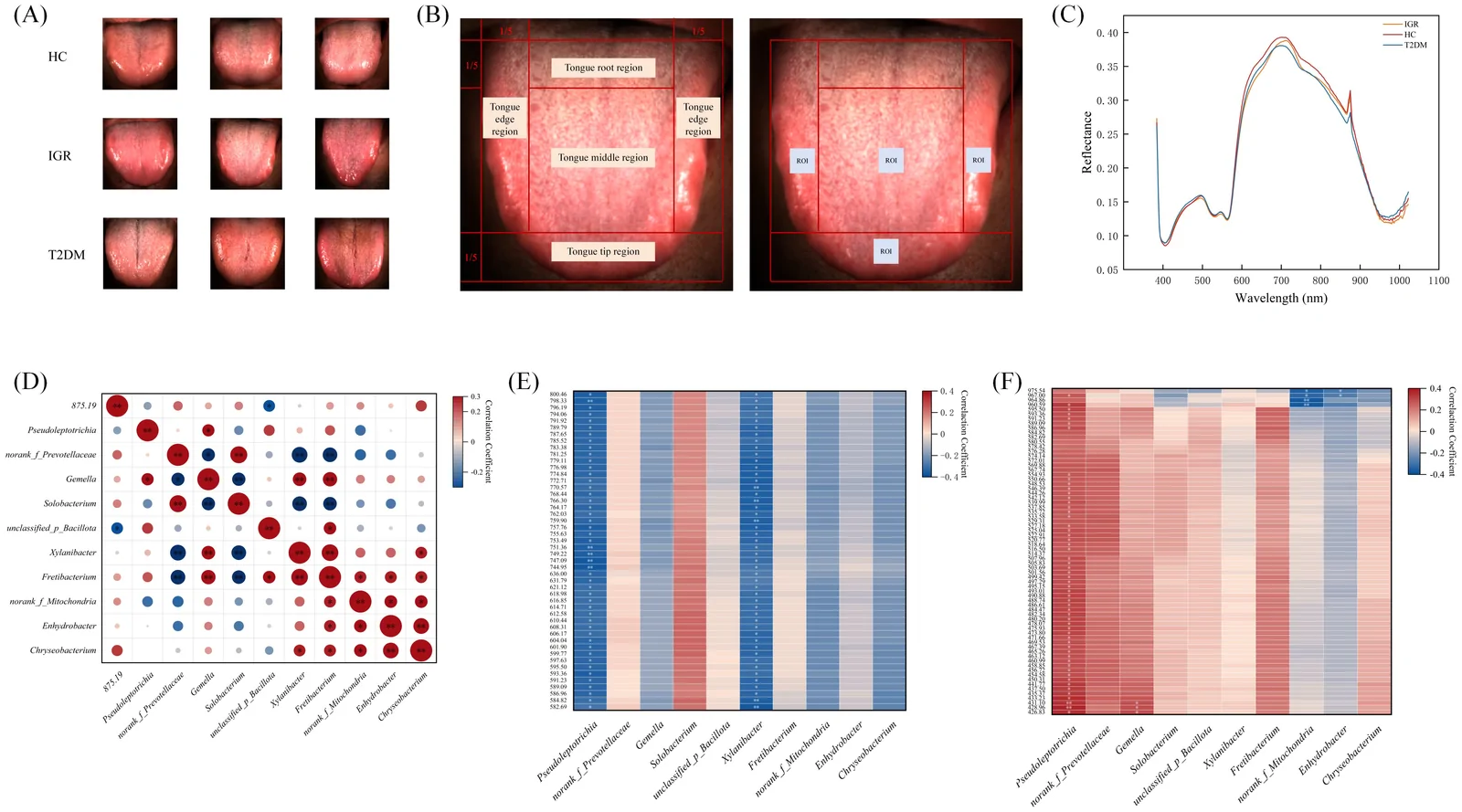
Hyperspectral tongue image analysis. **(A)** presents the hyperspectral pseudo-color images of HC, IGR, and T2DM participants; **(B)** presents the criteria for ROI selection. **(C)** presents the tongue surface hyperspectral curves of HC, IGR, and T2DM participants. **(D)** presents the correlation heatmap between hyperspectral tongue data and tongue coating microbiota in the tongue middle region; **(E)** presents the correlation heatmap between hyperspectral tongue data and tongue coating microbiota in the tongue tip region; **(F)** presents the correlation heatmap between hyperspectral tongue data and tongue coating microbiota in the left tongue region.

#### 2.5.3 Data preprocessing and feature extraction

All hyperspectral data analyses were performed using MATLAB R2022a. First, spectral data from all samples were standardized to eliminate the effects of scale differences among wavelength variables and variations in spectral intensity, ensuring that all spectral variables were transformed to a comparable statistical scale. Subsequently, the 153 subjects were randomly divided into training and independent testing sets at an 8:2 ratio using stratified random sampling. The training set was used for model development, parameter optimization, and internal validation, the independent test set was used to evaluate model generalization performance. Class labels were assigned as follows: HC were labeled as class 1, IGR as class 2, and T2DM as class 3. Due to the imbalance in sample size among the three groups, classification models may become biased toward the majority class, resulting in reduced recognition performance for minority classes. Therefore, synthetic minority oversampling technique (SMOTE) was applied to the training set for sample augmentation. SMOTE generates artificial samples by interpolating between minority class samples in the feature space, thereby improving the ability of classification models to learn minority class characteristics. After SMOTE processing, the training set was adjusted to 40 HC samples, 40 IGR samples, and 80 T2DM samples.

Considering that hyperspectral acquisition can be affected by the collection environment and equipment, multiple spectral preprocessing methods were applied to reduce noise and redundant information while improving the stability of spectral features. Including Savitzky-Golay (S-G) smoothing, first derivative (1^st^D), standard normal variate (SNV), and multiplicative scatter correction (MSC), as well as their combinations. To systematically assess the impact of different spectral preprocessing methods on classification performance, all preprocessing strategies were evaluated under the same modeling conditions. For each tongue region, the preprocessing method with the highest F1-score obtained from 5-fold-cross validation on the training set was selected as the optimal strategy and was then fixed before being applied to the independent test set for final model evaluation. S-G smoothing effectively reduces or eliminates random noise and baseline offset by fitting a specialized polynomial within a moving window, thereby enhancing the signal-to-noise ratio of the hyperspectral dataset while preserving critical spectral features and achieving data smoothing without compromising signal fidelity. 1^st^D is commonly used to correct additive baseline offsets in spectra by removing wavelength-independent baseline variations, thereby eliminating baseline drift and reducing background interference. This method yields clearer spectral curves while reflecting the variation trends of the original spectrum, and it also possesses a certain capacity for separating characteristic peaks affected by interference. SNV corrects spectra based on variance, which can effectively eliminate the effects of solid particle size, surface scattering, and path length variations on the spectra. By removing baseline drift and slope variations while reducing noise and improving the signal-to-noise ratio, SNV enhances the accuracy of spectral data. MSC is a least-squares-based spectral correction method that effectively eliminates light scattering and interference caused by uneven particle size distribution and differences in surface scattering properties among samples, thereby reducing spectral data errors and strengthening the correlation between spectra and data.

Hyperspectral data typically contain high-dimensional wavelength variables. Direct modeling using all wavelengths may increase model complexity and lead to overfitting. Therefore, partial least squares discriminant analysis (PLS-DA) was applied for dimensionality reduction. PLS-DA constructs latent variables (LVs) by maximizing the relationship between spectral variables and class information, thereby extracting discriminative spectral patterns while reducing variable redundancy. Based on variable contributions, informative spectral features with high discriminative capability were selected and subsequently used for classification model development.

#### 2.5.4 Machine learning model construction

Based on the preprocessed and selected hyperspectral features, three classification models, including SVM, RF, and PLS-DA, were developed to distinguish among HC, IGR, and T2DM groups. SVM is a commonly used supervised learning algorithm based on kernel function methods, frequently applied in classification and regression analysis. It classifies data by mapping low-dimensional nonlinear input variables into a high-dimensional linear space or by utilizing an optimal hyperplane. This model exhibits strong stability and is less affected by dimensionality and overfitting. The linear kernel function is characterized by computational simplicity and high efficiency; therefore, the linear kernel function was adopted for modeling analysis in this study. RF performs classification and regression by constructing multiple decision trees, thereby reducing model variance and overfitting risk. Simultaneously, it employs the remaining one-third of samples to calculate out-of-bag error for cross-validation and variable importance assessment. Models established based on the RF algorithm typically demonstrate high accuracy and robustness; however, compared with a single decision tree, the decision-making process of the model is less intuitively interpretable. In this study, the number of decision trees was set to 50, 100, 200, and 300, and the minimum leaf size was set to 1, 5, 10, and 20. PLS-DA is a supervised learning method that integrates dimensionality reduction and classification functions. By maximizing the covariance between spectral variables and class labels, PLS-DA constructs a latent variable space to achieve discrimination among different metabolic states.

All models were internally validated using 5-fold cross-validation. The training dataset was randomly divided into five subsets. In each iteration, four subsets were used for model training, and the remaining subset was used for validation. This process was repeated five times to obtain a robust estimation of model performance. During model training, parameter optimization was performed exclusively using the training dataset. After model training, the optimized models were applied to the independent test set. Classification performance was evaluated based on prediction results, and the performance differences among SVM, RF, and PLS-DA models were compared for the three-class classification task.

#### 2.5.5 Model evaluation

To comprehensively evaluate the classification performance of different machine learning models, multiple evaluation metrics were employed. Model performance was primarily assessed based on predictions from the independent test set, combined with cross-validation results from the training set to evaluate model stability.

The evaluation metrics included accuracy (ACC), precision, recall, specificity, F1-score, and area under the receiver operating characteristic curve (AUC). For each class, sensitivity, specificity, precision, and AUC values were calculated separately to assess the model’s ability to identify different metabolic states. ACC represents the proportion of correctly classified test samples relative to the total number of test samples, that is, the ratio of correctly classified positive cases to the total confirmed positive cases, and it measures the overall predictive performance of the model. Precision refers to the proportion of samples predicted as positive that are actually positive, reflecting the accuracy of the model’s predictions for positive samples. Recall refers to the proportion of actual positive samples that are correctly predicted as positive by the model, reflecting the model’s ability to correctly identify positive cases. Specificity, also known as the true negative rate, represents the probability of correctly indicating a non-diseased state; higher specificity corresponds to a greater likelihood of correct diagnosis. The F1 score is the harmonic mean of precision and recall, capable of comprehensively evaluating the overall performance of the model. AUC is the area under the receiver operating characteristic (ROC) curve, measuring the ability of a classification model to rank positive samples ahead of negative samples, with values ranging from 0.5 to 1; higher values indicate better classification performance.

Finally, ROC curves and confusion matrices were generated to visualize classification performance. The optimal model was selected based on comprehensive performance metrics obtained from the independent test set.

#### 2.5.6 Statistical analysis

Statistical analysis was performed using SPSS 21.0 software (IBM Corp, Armonk, NY, USA). Measurement data were first subjected to normality testing (Shapiro-Wilk test) and homogeneity of variance testing (Levene test). For data conforming to normal distribution with equal variances, results were expressed as mean ± standard deviation ( $\bar{\text{x}}$ ± s), and comparisons between two groups were conducted using independent samples t-test, while comparisons among multiple groups were performed using one-way analysis of variance (One-way ANOVA). For data not conforming to normal distribution or with unequal variances, results were expressed as median and interquartile range M (Q1, Q3), and comparisons between two groups were conducted using Wilcoxon rank-sum test, while comparisons among multiple groups were performed using Kruskal-Wallis rank-sum test. Enumeration data were described as frequency and percentage (n%), and intergroup comparisons were conducted using chi-square test or Fisher’s exact probability test. A *P* value less than 0.05 was considered statistically significant.

Correlation analysis was performed on hyperspectral tongue image characteristic wavelengths and tongue coating microorganisms that showed significant differences among the three groups. The Shapiro-Wilk test was first applied; Pearson correlation analysis was used for data conforming to normal distribution, and Spearman correlation analysis was used for data not conforming to normal distribution. A P value less than 0.05 was considered to indicate significant correlation.

## 3 Results

### 3.1 Comparison of baseline characteristics

In accordance with the ethical requirements of the Declaration of Helsinki and clinical routine practice, non-essential invasive blood tests, including lipid profiles, liver and renal function tests, and routine blood examinations, were not performed on HC participants. Furthermore, given the high rate of missing blood biochemical data in the IGR group, the following grouping strategy was adopted for baseline analysis in this study: the IGR group and T2DM participants with disease duration of less than 1 year were combined into the abnormal glucose metabolism group, while T2DM participants with disease duration of 1 year or more were classified into the T2DM group for comparison of blood biochemical indicators between the two groups. Non-invasive indicators including general demographic data were compared among the HC, IGR, and T2DM groups. The comparison results are presented in **Tables 1** and **2**.

**Table 1:** General characteristics of HC, IGR, and T2DM participants.

General information	HC	IGR	T2DM	*P*-value
Age (Years)	49.00 (41.00, 54.50)	50.50 (42.00, 61.00)	50.00 (42.00, 60.00)	0.179
Sex (Male/Female)	6/30	7/11	74/25	0.000
Smoking (0/1/2/3)	32/0/0/4	15/0/0/3	46/11/2/40	0.000
Drinking (0/1/2/3)	31/1/3/1	12/0/5/1	53/6/20/20	0.015
Family (0/1/2)	2/9/25	3/6/9	9/48/42	0.053
BMI (kg/m²)	24.45 (21.67, 26.56)	25.70 (22.89, 28.06)	26.87 (24.97, 29.16)	0.001
FBG (mmol/l)	5.20 (5.00, 5.40)	6.15 (6.10, 6.62)	6.90 (6.00, 7.90)	0.000
2 h PG (mmol/l)	6.40 (5.85, 6.50)	9.30 (8.80, 9.50)	10.15 (8.90, 13.48)	0.000

In smoking history: 0, never smoked; 1, quit smoking; 2, light smoker; 3, heavy smoker. In alcohol consumption history: 0, never drank; 1, quit drinking; 2, light drinker; 3, heavy drinker. In family history: 0, unknown; 1, positive family history of genetic disorders; 2, negative family history of genetic disorders.

**Table 2:** Clinical indexes of abnormal glucose metabolism participants and T2DM participants.

Clinical indexes	Abnormal glucose metabolism	T2DM	*P*-value
HbA1c (mmol/l)	7.00 (6.70, 7.45)	7.00 (6.50, 9.00)	0.761
TC (mmol/l)	4.82 ± 0.71	4.98 ± 1.23	0.690
TG (mmol/l)	2.00 (1.21, 2.61)	1.50 (1.07, 2.49)	0.472
HDL-C (mmol/l)	1.05 (0.91, 1.14)	0.96 (0.86, 1.09)	0.54
LDL-C (mmol/l)	2.95 ± 0.82	3.00 ± 0.90	0.892
ALT (U/L)	37.00 (26.00, 48.00)	25.00 (17.50, 44.00)	0.115
AST (U/L)	25.00 (19.50, 34.00)	19.00 (15.00, 25.00)	0.072
GGT (U/L)	47.00 (36.10, 73.70)	33.65 (25.60, 49.60)	0.092
ALP (U/L)	81.18 ± 18.99	71.67 ± 12.02	0.149
TP (G/L)	71.50 (68.70, 73.45)	70.75 (63.05, 74.55)	0.662
Alb (G/L)	44.69 ± 3.31	42.67 ± 4.51	0.192
Glb (G/L)	25.87 ± 5.41	26.90 ± 3.13	0.482
A/G (G/L)	1.79 ± 0.35	1.60 ± 0.21	0.058
Cr (Umol/L)	63.75 ± 9.21	66.81 ± 16.51	0.627
BUN (mmol/l)	5.55 ± 1.35	6.49 ± 1.77	0.188
UA (Umol/L)	338.25 ± 57.28	326.65 ± 114.11	0.787
IV-Col (ng/mL)	75.16 ± 15.25	97.00 ± 26.27	0.008
WBC (10 ^ 9/L)	6.76 ± 1.39	6.19 ± 1.21	0.289
RBC (10 ^ 12/L)	5.17 ± 0.57	4.86 ± 0.63	0.242
HCT (%)	46.13 ± 3.57	43.62 ± 4.88	0.201
HGB (g/l)	157.00 ± 14.40	148.15 ± 17.17	0.21
CRP (mg/L)	1.12 (0.90, 1.45)	2.46 (1.65, 2.83)	0.019
Glu (0/1/2/3/4)	5/2/0/2/2	9/1/1/4/14	0.259
WBC (0/1/2/3)	9/0/1/1	24/3/2/0	0.207
Pro (0/1/2/3)	9/1/0/1	23/3/2/1	0.624
BLD (0/1/2/3)	8/2/1/0	24/1/3/1	0.432
SG (G/ml)	1.03 (1.02, 1.03)	1.02 (1.01, 1.03)	0.165
pH	5.00 (5.00, 5.25)	5.50 (5.00, 6.00)	0.091

For Glu: 0, -; 1, +; 2, ++; 3, +++; 4, ++++. For WBC: 0, -; 1, ±; 2, +; 3, ++. For Pro: 0, -; 1, ±; 2, +; 3, +++. For BLD: 0, -; 1, ±; 2, +; 3, ++.

A total of 153 participants were enrolled in this study, comprising 36 HC, 18 IGR, and 99 T2DM participants. As shown in **Table 1**, no statistically significant difference in age was observed among the three groups (*P* > 0.05), indicating comparability. Statistically significant differences were found in gender distribution, smoking history, alcohol consumption history, and BMI among the three groups (*P* < 0.05). The proportion of male participants and BMI levels were higher in the T2DM group than in the HC and IGR groups, and significant differences in smoking and alcohol consumption status were observed among the three groups.

To further evaluate the potential influence of these confounding factors, multinomial logistic regression analysis was performed for adjustment (**Supplementary Table 1**). Using the HC group as the reference category, age was significantly positively associated with both IGR and T2DM status. In the IGR group, each one-year increase in age was associated with a 7.3% increase in the risk of IGR (OR = 1.073, 95% CI: 1.008-1.142, *P* = 0.027). In the T2DM group, both age (OR = 1.078, 95% CI: 1.025-1.134, *P* = 0.004) and BMI (OR = 1.170, 95% CI: 1.022-1.340, *P* = 0.023) were independently associated with T2DM status. Sex was significantly associated with T2DM status, with male participants showing a significantly higher likelihood of T2DM compared with female participants (OR = 12.667, 95% CI: 3.106-51.654, *P* < 0.001). The association between sex and IGR status did not reach statistical significance (OR = 3.989, 95% CI: 0.696-22.864, *P* = 0.120). Neither smoking history nor alcohol consumption history showed significant associations with IGR or T2DM status (all *P* > 0.05). Therefore, age was identified as a common factor associated with both IGR and T2DM, whereas higher BMI and male sex were independently associated primarily with T2DM status.

### 3.2 Analysis of tongue coating microorganisms

#### 3.2.1 Alpha diversity analysis

A total of 53 tongue coating samples were subjected to 16S rRNA gene sequencing, followed by splicing, filtering, and tag evaluation, ultimately yielding 1109 operational taxonomic units (OTUs), as shown in **Figure 3A**. Among the three groups, 525 OTUs were shared in common; the HC group possessed 128 unique OTUs; the IGR group possessed 68 unique OTUs; and the T2DM group possessed 201 unique OTUs, with the T2DM group exhibiting the highest number of OTUs.

**Figure 3:**
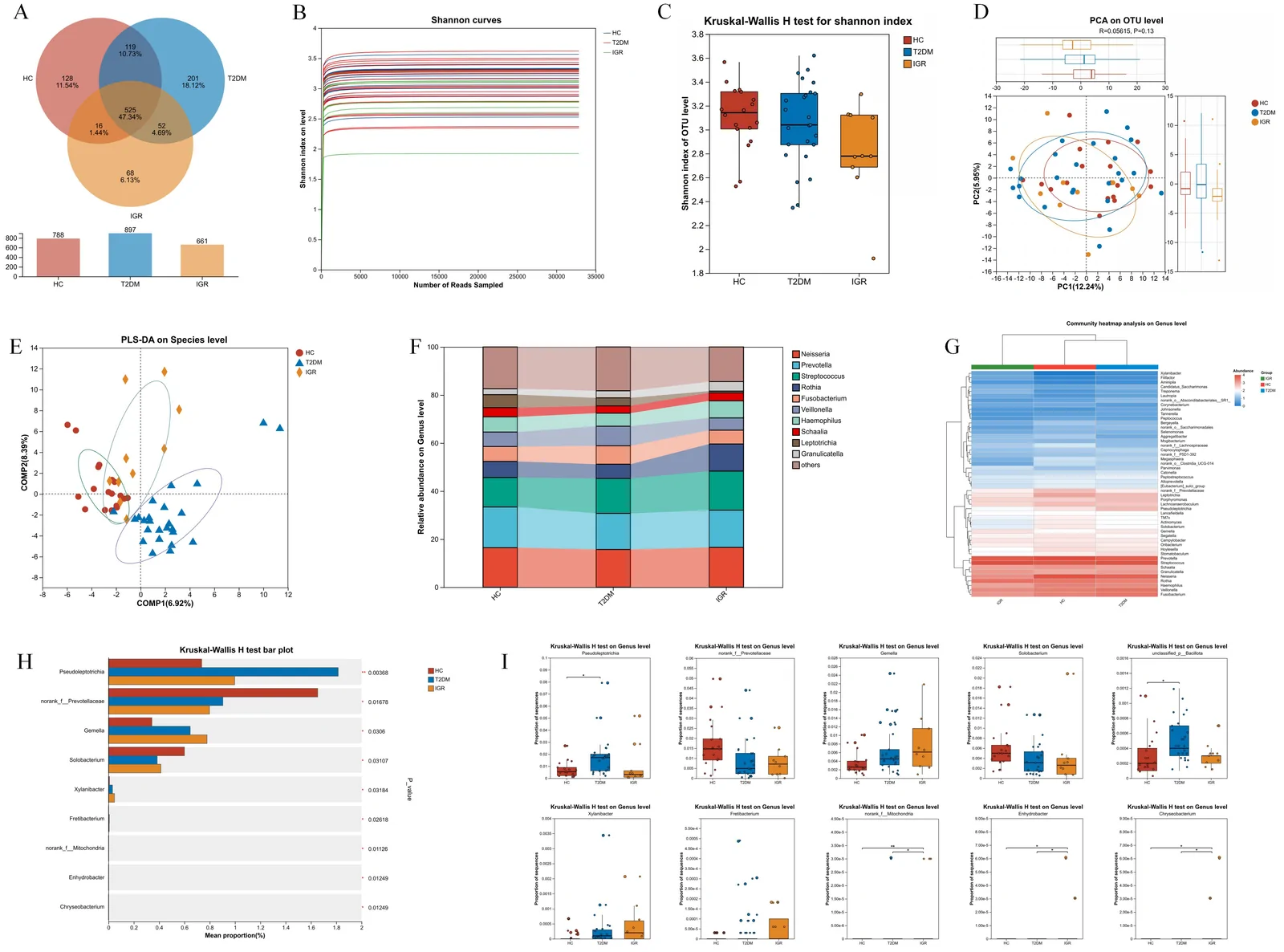
Microbiota analysis. **(A)** presents the Venn diagram of OTU distribution; **(B)** presents the rarefaction curve; **(C)** presents the box plot of intergroup difference testing for alpha diversity indices; **(D)** presents the PCA plot of tongue coating microorganisms; **(E)** presents the PLS-DA plot of tongue coating microorganisms; **(F)** presents the stacked bar chart of the top 10 tongue coating microbial communities at the genus level; **(G)** presents the heatmap of tongue coating microbial communities at the genus level; **(H)** presents the bar chart of differential testing for tongue coating microorganisms at the genus level; **(I)** presents the box plot of significance testing for differential tongue coating microorganisms at the genus level.

In this study, rarefaction curves combined with alpha diversity indices were employed to evaluate whether the sequencing depth adequately covered the microorganisms present in the samples, providing an intuitive reflection of the rationality of the sequencing data volume. As shown in **Figure 3B**, higher Shannon index values indicate greater diversity of the oral microbiota. All 53 tongue coating samples plateaued after reaching a sequencing depth of 10,000 reads, indicating that the sequencing data volume was sufficient to cover the microbial diversity of each sample and that the subsequent analysis results were reliable.

To assess bacterial community diversity, the Shannon index was used for intergroup comparison in this study, and statistical analysis was performed using the Kruskal-Wallis H non-parametric test. As shown in **Figure 3C**, the median Shannon index of the HC group was approximately 3.15, with a relatively concentrated distribution, suggesting that the oral microbiota diversity in healthy populations is relatively stable. The median Shannon index of the T2DM group was slightly lower than that of the HC group, but the data showed the greatest degree of dispersion, indicating significant individual variation in microbiota diversity among T2DM patients, with some patients exhibiting markedly reduced diversity. The IGR group showed the lowest median value, suggesting a downward trend in microbiota diversity among the IGR population. No statistically significant differences in Shannon index were observed among the three groups (*P* > 0.05), indicating that although the oral microbiota diversity in T2DM and IGR participants showed a declining trend, the richness did not decrease significantly. Furthermore, a *post hoc* power analysis was conducted to evaluate the statistical power of the alpha diversity comparison among the three groups. Based on the observed effect size (Cohen’s f = 0.3702) and an alpha level of 0.05, approximately 25 participants per group would be required to achieve 80% statistical power. The current cohort included 18 HC participants, 10 IGR participants, and 25 T2DM participants. These results suggest that the current sample size was sufficient to detect relatively large differences in alpha diversity but may have limited power to identify smaller effects among the three groups.

#### 3.2.2 Beta diversity analysis

Beta diversity was employed in this study to evaluate the differences in community structure among tongue coating samples from the HC, IGR, and T2DM groups, and the effectiveness of grouping was tested through analysis of similarities (ANOSIM). As shown in **Figure 3D**, R > 0 indicated that intergroup differences were greater than intragroup differences, confirming the validity of the grouping. Principal components analysis (PCA) based on the OTU level was used to assess the differences in oral microbiota structure among the HC, IGR, and T2DM groups. As shown in **Figure 3D**, the first principal component (PC1) explained 12.24% of the microbiota variation, and the second principal component (PC2) explained 5.85%, with a cumulative explanatory rate of 18.09%. Based on the scatter plot distribution, the overall structure of the oral microbiota among the three groups did not exhibit significant intergroup differences at the OTU level, and further analysis is required to reveal potential differences in microbiota structure.

To further investigate the differences in oral microbiota structure at the species level among the HC, IGR, and T2DM groups, PLS-DA was employed for beta diversity analysis in this study, as shown in **Figure 3E**. The first component (COMP1) explained 6.92% of the microbiota variation, and the second component (COMP2) explained 8.39%, with a cumulative explanatory rate of 15.31%. In the two-dimensional score plot constructed by COMP1 and COMP2, the three groups demonstrated a clear trend of separation. However, a certain degree of overlap was observed among the confidence ellipses of the three groups, indicating that some similarity in microbiota structure still existed among the groups. Compared with PCA, PLS-DA exhibited stronger intergroup discriminatory capability at the species level, suggesting that with the aggravation of glucose metabolism disorder, the species composition structure of the oral microbiota undergoes characteristic alterations.

#### 3.2.3 Analysis of relative abundance of tongue coating microbiota

To visually demonstrate the structural characteristics of oral microbiota composition at the genus level among the HC, IGR, and T2DM groups, community stacked bar charts were employed in this study for visual analysis of the distribution of major dominant genera. As shown in **Figure 3F**, the figure presents the compositional proportions of the top 10 genera ranked by relative abundance in each group as percentages. The results revealed that the oral microbiota of the three groups at the genus level was composed of core genera including *Neisseria, Prevotella, Streptococcus, Rothia, Fusobacterium, Veillonella, Haemophilus, Schaalia, Leptotrichia*, and *Granulicatella*.

Community heatmaps were used in this study for visual analysis of the relative abundance of 50 genera, as shown in **Figure 3G**. Based on the color gradient distribution, three abundance clusters could be distinguished. The first cluster, comprising genera from *Xylanibacter* to *[Eubacterium]_sulci_group*, maintained relatively low relative abundance across all three groups. The second cluster, including genera from *norank_f:Prevotellaceae* to *Stomatobaculum*, exhibited characteristics of medium abundance distribution in the three groups. The third cluster, ranging from *Prevotella* to *Fusobacterium*, demonstrated high abundance distribution in all three groups, constituting the core oral microbiota. This figure revealed that certain differences existed in the oral microbial community structure among the IGR, HC, and T2DM groups.

To systematically evaluate the statistical differences in oral microbiota composition among the HC, IGR, and T2DM groups, the Kruskal-Wallis H test was employed for multi-group comparative analysis, as shown in **Figure 3H**. A total of 10 genera exhibited statistically significant differences (*P* < 0.05), including *Pseudoleptotrichia, norank_f_Prevotellaceae, Gemella, Solobacterium,unclassified_p_Bacillota, Xylanibacter, Fretibacterium, norank_f_Mitochondria, Enhydrobacter*, and *Chryseobacterium*. Specifically, *Pseudoleptotrichia* and *unclassified_p_Bacillota* demonstrated significantly higher relative abundance in the T2DM group compared with the HC and IGR groups, showing a disease-enrichment trend. In contrast, *norank_f_Prevotellaceae* and *Solobacterium* were significantly higher in the HC group than in the IGR and T2DM groups, gradually decreasing with the aggravation of glucose metabolism disorder. *Gemella*, *Xylanibacter*, and *Fretibacterium* decreased in the HC group but were most abundant in the IGR group, exhibiting an elevated intermediate state, suggesting that the proliferation of these low-abundance genera may be associated with glucose metabolism disorder. *norank_f_Mitochondria*, *Enhydrobacter*, and *Chryseobacterium* were not detected in the HC group but were detected in small quantities in the IGR or T2DM groups. Although their absolute abundance was extremely low, this distribution pattern from absence to presence suggests that these genera may participate in the pathological process of glucose metabolism disorder as opportunistic pathogens, and their biological significance requires further validation combined with functional analysis. Box plot analysis (**Figure 3I**) further revealed that *Pseudoleptotrichia* and *norank_f_Prevotellaceae* exhibited the most pronounced separation trends among the three groups, with relatively small intragroup variation, suggesting that these two genera possess good potential for intergroup discrimination. In contrast, genera such as *Gemella* and *Solobacterium* showed greater intragroup dispersion, with some samples presenting exceptionally high values, reflecting the individual variability in microbiota composition. In summary, characteristic differences in microbiota composition at the genus level exist among the three groups, and these differential genera are expected to serve as potential microbial markers for distinguishing populations with different glucose metabolism states, providing novel targets for early diagnosis and risk assessment of type 2 diabetes and impaired glucose regulation.

### 3.3 Average spectra of hyperspectral tongue images

In this study, hyperspectral images were extracted from 36 HC participants, 18 IGR participants, and 99 T2DM participants. **Figure 2A** presents representative hyperspectral pseudo-color images from the three groups. Four ROIs were selected from the middle of the tongue, the tongue tip, the left side of the tongue, and the right side of the tongue, and the average spectra of each region within the spectral range of 380~1030 nm were extracted. To reflect the integrity of the tongue image, the average spectra of the aforementioned four ROIs were further calculated to construct the tongue surface spectral features. The tongue surface can comprehensively reflect the overall optical characteristics of the tongue image and is more complete than a single region; therefore, we compared the average spectra of the tongue surface to investigate the differences in spectral features of tongue images under abnormal glucose metabolism states.

The spectral curves of the tongue surface exhibited typical tissue reflectance characteristics. As shown in **Figure 2C**, the spectral curves of the three groups demonstrated generally consistent trends in the visible to near-infrared range, although differences were observed at specific bands. In the visible region (380~780 nm), the overall reflectance increased with increasing wavelength, with absorption valleys near 400 nm, 530 nm, and 565 nm, and reflectance peaks at 500 nm, 545 nm, and 700 nm. In the near-infrared region (780~1030 nm), the reflectance gradually decreased, but an obvious peak appeared at 870 nm and valley formed at 865 nm and 970 nm.

Intergroup comparison revealed that in the visible light range of 380~540 nm, the reflectance followed the pattern of T2DM group > HC group > IGR group; in the range of 540~680 nm, the reflectance followed the pattern of HC group > T2DM group > IGR group; in the range of 680~750 nm, the differences among the three groups were most significant, with HC group > IGR group > T2DM group; in the range of 750~950 nm, the curves of the three groups gradually converged, showing a trend of HC group > IGR group > T2DM group; near 950~1030 nm, the pattern was T2DM group > HC group > IGR group.

The average spectral curves of the tongue surface intuitively reflected the spectral characteristics of tongue images in populations with different glucose metabolism states, providing important spectroscopic evidence for subsequent objective research on T2DM tongue diagnosis based on hyperspectral technology. The difference in the main peak at 700 nm is expected to serve as a potential spectral marker for distinguishing different glucose metabolism states.

### 3.4 Correlation analysis between hyperspectral tongue data and tongue coating microbiota

Based on the middle of the tongue, tongue tip, left side of the tongue, and right side of the tongue regions of HC, IGR, and T2DM participants, Spearman correlation analysis was performed between hyperspectral data at wavelengths with significant differences and the relative abundance of 10 key tongue coating microbial genera. The correlation coefficients and their significance between hyperspectral tongue data and tongue coating microorganisms were visualized through a correlation heatmap, as shown in **Figures 2D–F**.

In the middle tongue region, *unclassified_p_Bacillota* showed a negative correlation with spectral reflectance at 875.19 nm (*r* = -0.285, *P* < 0.05), suggesting that as *unclassified_p_Bacillota* abundance increased, reflectance in the middle tongue region concomitantly decreased. In the tongue tip region, *Pseudoleptotrichia* demonstrated negative correlations with spectral reflectance at wavelengths of 582.69~621.12 nm, 631.79 nm, 636 nm, and 744.95~800.46 nm (-0.374 < *r* < -0.302, *P* < 0.05), with the strongest negative correlation observed at 744.95 nm (*r* = -0.374, *P* < 0.01). Similarly, *Xylanibacter* showed negative correlations with spectral reflectance at 582.69~621.12 nm, 631.79 nm, 636 nm, and 744.95~800.46 nm (-0.367 < *r* < -0.273, *P* < 0.05), with the strongest negative correlation at 582.69 nm (*r* = -0.367, *P* < 0.01). These findings suggest that increased abundance of *Pseudoleptotrichia* and *Xylanibacter* was accompanied by decreased spectral reflectance in the tongue tip region. In the left tongue region, *Pseudoleptotrichia* abundance showed positive correlations with spectral reflectance at wavelengths of 426.83~437.50 nm, 441.77 nm, 450.31 nm, 454.58~507.96 nm, 516.50~527.15 nm, 531.45~550.66 nm, 554.93 nm, 586.96~595.50 nm, and 967.00 nm (0.271 < *r* < 0.364, *P* < 0.05), with the strongest positive correlation at 428.96 nm (*r* = 0.364, *P* < 0.01). *Gemella* abundance showed positive correlations with spectral reflectance at 426.83~431.10 nm (0.281 < *r* < 0.293, *P* < 0.05). These findings suggest that increased abundance of *Pseudoleptotrichia* and *Gemella* was accompanied by elevated spectral reflectance in the left tongue region. In contrast, *norank_f_Mitochondria* abundance showed negative correlations with spectral reflectance at 960.59 nm, 964.86~967.00 nm, and 975.54 nm (-0.374 < *r* < -0.341, *P* < 0.05), with the strongest negative correlations at 960.59 nm and 964.86 nm (*r* = -0.374, *P* < 0.01). *Enhydrobacter* abundance showed negative correlations with spectral reflectance at 967.00 nm and 975.54 nm (-0.285 < *r* < -0.278, *P* < 0.05). These findings suggest that increased abundance of *norank_f_Mitochondria* and *Enhydrobacter* was accompanied by decreased spectral reflectance in the left tongue region. However, after Benjamini–Hochberg false discovery rate (BH-FDR) correction, none of these associations remained statistically significant (adjusted *q* > 0.05), indicating that these findings should be interpreted as exploratory associations requiring validation in larger cohorts.

### 3.5 Results of T2DM disease status identification models

To determine the optimal spectral preprocessing strategy, the effects of different preprocessing methods on machine learning classification performance were systematically evaluated. Spectra without additional preprocessing (None), S-G smoothing, 1^st^D, SNV, MSC, and their combinations were compared. Model performance was comprehensively evaluated using Acc, recall, specificity, F1 score, and AUC (**Supplementary Tables 2**–**6**). Different spectral preprocessing methods significantly affected model classification performance. Across different tongue regions, the SVM-based classification models consistently achieved superior overall performance after S-G smoothing. The S-G smoothing preprocessing method effectively improved model classification capability and was therefore selected as the optimal spectral preprocessing strategy for subsequent model development. Subsequently, PLS-DA was applied to reduce the dimensionality of the spectral data, and the optimal number of latent variables (LVs) was determined based on model performance. The optimal numbers of LVs for different tongue regions were 18 for the tongue middle region, 25 for the tongue tip region, 15 for the left tongue edge region, 25 for the right tongue edge region, and 20 for the whole tongue surface region.

To further investigate the contribution of different tongue regions to disease state discrimination, classification models were established using spectral features extracted from the tongue middle, tongue tip, left tongue edge, right tongue edge, and tongue surface regions, respectively. As shown in **Table 3**, substantial differences in classification performance were observed among different tongue regions for the three-class classification task involving HC, IGR, and T2DM. Among all regions, the SVM model based on the tongue middle region exhibited the best performance on the independent test set, achieving an ACC of 0.89655 and an AUC of 0.80290. In comparison, the ACC of models based on the tongue tip, left tongue edge, and right tongue edge regions were 0.79310, 0.75862, and 0.72414, respectively, all lower than that of the tongue middle region model. Although the tongue surface regions model incorporated more spatial information, its accuracy (0.82759) did not exceed that of the tongue middle region model. These findings suggest that the tongue middle region may contain more stable and discriminative spectral information associated with metabolic disease states. The discriminative capability of the SVM model based on spectral features from the tongue middle region was further evaluated for distinguishing among HC, IGR, and T2DM groups. As shown in **Table 4**, For the HC group, the model achieved high classification performance (F1-score = 0.92308, AUC = 0.87662). For the T2DM group, the model exhibited robust classification performance (F1-score = 0.95000, AUC = 0.85263). For the IGR group, the classification performance was relatively limited. (F1-score = 0.40000, AUC = 0.67949).

**Table 3:** Performance evaluation of SVM classification models based on spectral features from different tongue regions in the independent test set.

Region	Model	ACC	Precision	Recall	Specificity	F1-Score	AUC
Tongue middle	SVM	0.8966	0.8016	0.7302	0.9205	0.7577	0.8029
Tongue tip	SVM	0.7931	0.8609	0.6174	0.8182	0.6746	0.7054
Tongue left	SVM	0.7586	0.6691	0.7235	0.8622	0.6905	0.7774
Tongue right	SVM	0.7241	0.6429	0.5823	0.7902	0.6051	0.8029
Tongue surface	SVM	0.8276	0.7302	0.6650	0.8720	0.6897	0.8681

**Table 4:** Class-wise classification performance of SVM models based on hyperspectral features from different tongue regions in the independent test set.

Region	Class	Precision	Recall	Specificity	F1_score	AUC
Tongue middle	HC	1.0000	0.8571	1.0000	0.9231	0.8766
	IGR	0.5000	0.3333	0.9615	0.4000	0.6795
	T2DM	0.9048	1.0000	0.8000	0.9500	0.8526
Tongue tip	HC	0.8000	0.5714	0.9546	0.6667	0.7857
	IGR	1.0000	0.3333	1.0000	0.5000	0.6410
	T2DM	0.7826	0.9474	0.5000	0.8571	0.6895
Tongue left	HC	0.6250	0.7143	0.8636	0.6667	0.8377
	IGR	0.5000	0.6667	0.9231	0.5714	0.9359
	T2DM	0.8824	0.7895	0.8000	0.8333	0.8211
Tongue right	HC	0.6667	0.5714	0.9091	0.6154	0.8182
	IGR	0.5000	0.3333	0.9615	0.4000	0.8462
	T2DM	0.7619	0.8421	0.5000	0.8000	0.7737
Tongue surface	HC	0.8333	0.7143	0.9546	0.7692	0.8701
	IGR	0.5000	0.3333	0.9615	0.4000	0.8974
	T2DM	0.8571	0.9474	0.7000	0.9000	0.8368

ROC curves and confusion matrices were further generated to visually evaluate the performance of the optimal SVM model. As shown in **Figure 4** the model demonstrated discriminative capability for all three classes. Confusion matrix analysis indicated that most HC and T2DM samples were correctly classified, whereas several IGR samples were misclassified. It demonstrated effective discrimination among HC, IGR, and T2DM groups and supported the feasibility of hyperspectral tongue imaging for identifying abnormal glucose metabolism states.

**Figure 4:**
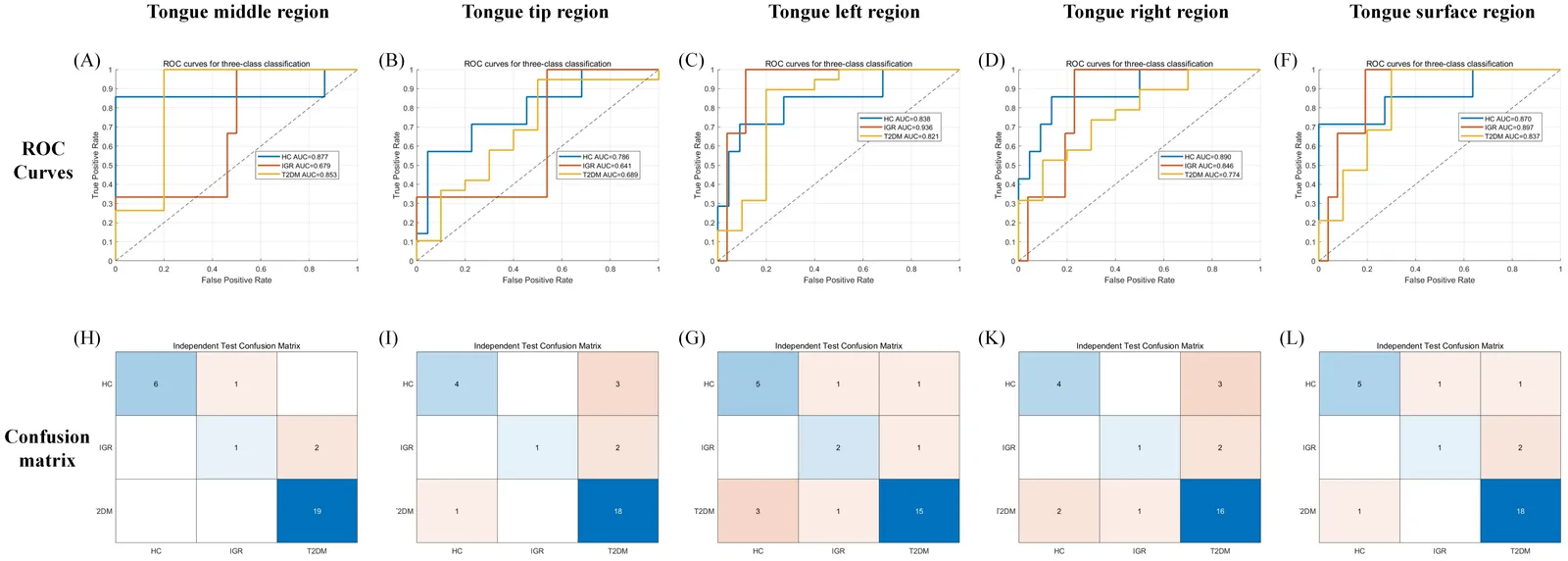
ROC curves and confusion matrices. **(A)** presents the ROC curve constructed by SVM combined with S-G smoothing preprocessing for the tongue middle region; **(B)** presents the ROC curve constructed by SVM combined with S-G smoothing preprocessing for the tongue tip region; **(C)** presents the ROC curve constructed by SVM combined with S-G smoothing preprocessing for the tongue left region; **(D)** presents the ROC curve constructed by SVM combined with S-G smoothing preprocessing for the tongue right region; **(E)** presents the ROC curve constructed by SVM combined with S-G smoothing preprocessing for the tongue surface region. **(H)** presents the confusion matrix of the test set for the tongue middle region. **(I)** presents the confusion matrix of the test set for the tongue tip region. **(G)** presents the confusion matrix of the test set for the tongue left region. **(K)** presents the confusion matrix of the test set for the tongue right region. **(L)** presents the confusion matrix of the test set for the tongue surface region.

## 4 Discussion

A total of 153 participants were enrolled in this study, including 36 HC participants, 18 IGR patients, and 99 T2DM patients. As shown in **Table 1**, the three groups were comparable in age (*P* > 0.05), whereas the proportion of males, smoking history, alcohol consumption history, and BMI in the T2DM group were significantly higher than those in the HC and IGR groups (*P* < 0.05). Multinomial logistic regression analysis was further performed to adjust for potential confounding factors, including age, sex, BMI, smoking, and alcohol consumption. The results showed that age was independently associated with both IGR and T2DM status, whereas increased BMI and male sex were primarily associated with T2DM status. These findings are consistent with previous epidemiological studies reporting that advanced age, obesity, and male sex are associated with an increased risk of T2DM. Biochemical indicators revealed that FBG and 2h-PG were significantly elevated in the T2DM group (*P* < 0.05). Type IV collagen (IV-Col), as a marker of hepatic fibrosis, and CRP, as an inflammatory indicator, were significantly elevated in the T2DM group (*P* < 0.05), suggesting that with disease progression, fibrosis and inflammatory responses are aggravated in T2DM patients. Elevated IV-Col levels indicate that T2DM patients may have progression of metabolic-associated fatty liver disease. Multiple studies have demonstrated that diabetes is associated with the progression of hepatic fibrosis. Poustchi, H. et al. (26) identified obesity, elevated liver enzymes, and insulin resistance as key risk factors. The difference in CRP levels reflects the low-grade inflammatory state in patients with mid-to-late stage T2DM. Tan, K. C. et al. (27) found that elevated serum concentrations of AGEs in T2DM patients were independent determinants of plasma CRP levels, suggesting that the low-grade inflammatory state may originate from AGE-activated inflammatory responses. Brahimaj, A. et al. (28) confirmed that CRP is associated with the development of prediabetes and diabetes (HR = 1.26, 1.32). In addition, CRP is also associated with the progression of vascular lesions and diabetic foot.

T2DM disrupts oral homeostasis by reducing salivary secretion and increasing carbohydrate levels in the parotid gland, thereby promoting the growth of specific bacterial species. In this study, 16S rRNA gene sequencing was performed on 53 tongue coating samples to systematically analyze the diversity characteristics and compositional differences of oral microbiota among the HC, IGR, and T2DM groups. The results revealed that at the genus level, samples from the three groups were predominantly composed of core genera including *Neisseria, Prevotella, Streptococcus, Rothia, Fusobacterium, Veillonella, Haemophilus, Schaalia, Leptotrichia*, and *Granulicatella*, consistent with findings from previous studies. Chen, B. et al. (29) conducted 16S rRNA sequencing on T2DM and HC participants and found that *Neisseria* was the most abundant genus in the T2DM group, with *Haemophilus* and *Streptococcus* also exhibiting elevated abundance in diabetic patients. Agarwal, J. et al. (30) performed metagenomic sequencing of saliva from T2DM patients and found that *Prevotella*, *Streptococcus*, *Veillonella*, and *Leptotrichia* were significantly elevated in T2DM patients. Previous research has demonstrated that *Neisseria* is associated with nitrate reduction (31), oral *Neisseria* can reduce nitrate to nitrite, which is subsequently swallowed and converted to NO through enzymatic and non-enzymatic processes in the human body. As a gaseous signaling molecule, NO is essential for metabolic, neurological, cardiovascular health, and skeletal muscle function (32). *Prevotella*, *Streptococcus*, *Rothia*, *Fusobacterium*, *Veillonella*, *Haemophilus*, and *Schaalia* also participate in the nitrate reduction process (31). *Prevotella* possesses the potential to colonize the distal gastrointestinal tract (33), oral *Prevotella* can be detected in feces, and oral and intestinal strains within the same host frequently exhibit high homology, suggesting that the oral-gut axis may participate in regulating human health and disease states. *Streptococcus* produces acidic substances (34), lowering local oral pH and potentially increasing the risk of periodontal diseases and dental caries. Periodontal pathogens contain multiple virulence factors such as LPS, endotoxins, and lipoteichoic acids, which activate the TLR4/NF-κB pathway through interactions with mononuclear phagocytes, inducing the release of inflammatory cytokines including IL-1β, PGE2, TNF-α, and IL-6, thereby triggering inflammatory responses, reducing insulin-mediated glucose uptake, and generating systemic insulin resistance, ultimately leading to difficulty in glycemic control (35). *Prevotella (*36), *Fusobacterium (*37), *Leptotrichia*, and *Granulicatella (*38) are also associated with periodontal inflammation induced by pH reduction. *Fusobacterium* belongs to the family *Fusobacteriaceae*; Li, Y. et al. (39) explored the potential mechanisms of the oral-gut microbiota axis and found that increased abundance of oral *Fusobacteriaceae* in hyperglycemic patients may induce oral-gut axis dysregulation, subsequently leading to increased intestinal *Lactobacillus* and potentially causing myocardial damage. *Veillonella* belongs to the family *Veillonellaceae* and is a Gram-negative anaerobic coccus, representing one of the important pathogens responsible for severe infections; in T2DM patients, it can cause decubitus infections (40) and bacteremia (41). *Schaalia* belongs to the family *Actinomycetaceae*, while *Saccharibacteria* (TM7) is a class of symbiotic ultramicrobacteria that must depend on host bacteria for survival; *Schaalia odontolytica* serves as the primary host bacterium for TM7, providing the necessary living environment and nutrients. Bachtiar, B. M. et al. (42) evaluated the abundance changes of TM7 and its host *S. odontolytica* in saliva and subgingival biofilms of T2DM patients and found that TM7 and other investigated bacteria exhibited the highest abundance in T2DM patients without periodontal disease, gradually decreasing with the progression of periodontal inflammation, suggesting that TM7 may exert protective effects by inhibiting the pathogenicity of *S. odontolytica*. TM7 and *S. odontolytica* were positively correlated in saliva but significantly negatively correlated in subgingival biofilms of T2DM patients with periodontitis, indicating that the periodontal inflammatory environment may disrupt their original symbiotic relationship.

Further differential analysis revealed that 10 genera, namely *Pseudoleptotrichia*, *norank_f_Prevotellaceae*, *Gemella*, *Solobacterium*, *unclassified_p_Bacillota*, *Xylanibacter*, *Fretibacterium*, *norank_f_Mitochondria*, *Enhydrobacter*, and *Chryseobacterium*, showed statistically significant intergroup differences (*P* < 0.05). *Pseudoleptotrichia* is a newly separated genus from *Leptotrichia* in 2020 and was significantly enriched in the T2DM group. Wang, R. et al. (43) performed 16S rRNA gene sequencing analysis on saliva samples from Chinese elderly individuals and found that *Leptotrichia*, *Staphylococcus*, *Catonella*, and *Bulleidia* were significantly elevated in the oral cavity of T2DM patients. Among these, *Leptotrichia*, as a pathogen of periodontitis, forms a bidirectional regulatory relationship with the occurrence and development of T2DM. *Gemella* belongs to *Streptococcaceae* and was significantly elevated in both the T2DM and IGR groups. Murmu, M. et al. (38) found that *Gemella* possesses the ability to metabolize carbohydrates and belongs to glycolytic lactic acid-producing microorganisms. It is strongly positively correlated with *Granulicatella*, forming a metabolic symbiotic complex that is significantly enriched in T2DM. *Solobacterium* belongs to *Erysipelotrichaceae* and is a class of anaerobic bacteria capable of producing volatile sulfur compounds; its proliferation is closely associated with oral malodor (44). In this study, this genus exhibited higher abundance in the HC group, which may be related to the dietary habits and oral hygiene status of the sampled population. *unclassified_p_Bacillota* is a class of bacterial groups that has not yet been fully identified; *Bacillota* was formerly known as *Firmicutes*, and this genus showed significantly elevated abundance in the T2DM group, consistent with the meta-analysis results of Huang, M. et al. (45) on 34 studies of T2DM oral microbiota, which found that 14 studies consistently demonstrated elevated *Firmicutes* in T2DM patients. *Xylanibacter* belongs to *Prevotellaceae* and possesses the ability to decompose hemicellulose, pectin, and storage polysaccharides from plant cell walls (46). In this study, *Xylanibacter* abundance was elevated in both the T2DM and IGR groups, with the most pronounced increase observed in the IGR group, suggesting that dietary structure may differ among the three groups of participants, and the relationship between this genus and T2DM requires further investigation.

By comparing the spectral curves of the tongue surface among the three groups, we found that the overall trends of the spectral curves were essentially consistent, and the band of 680~750 nm was the most sensitive for discriminating the degree of abnormal glucose metabolism, providing spectroscopic evidence for the objective assessment of tongue diagnosis in diabetes and prediabetes. The tongue surface is richly vascularized, and light absorption is influenced by HBG. Previous studies have indicated that wavelengths around 500~600 nm are associated with HBG content. In this study, light reflectance in the range of 500~600 nm followed the pattern of T2DM group > IGR group > HC group. Laboratory examination results showed that the mean HBG value in individuals with abnormal glucose metabolism was 157.00 g/L, while that in diabetic participants was 148.15 g/L, consistent with previous findings that HGB content exhibits a declining trend in diabetic patients (47, 48). This finding aligns with the optical principle that higher hemoglobin content corresponds to lower tissue reflectance.

Although exploratory correlations were observed between tongue coating microbiota and hyperspectral reflectance features, the underlying biological mechanisms remain to be further elucidated. The hyperspectral reflectance characteristics of tongue coating are unlikely to be determined by individual bacterial taxa alone, but rather by the combined effects of microbial communities, their metabolites or biofilm structures, and the physiological status of the underlying tongue mucosal tissues. Previous studies have demonstrated that tongue coating color, thickness, and moisture content can substantially influence reflected spectra, particularly in the 500~600 nm wavelength range, indicating that the physical properties of the tongue coating surface are important determinants of tongue spectral signals. In addition, optical reflectance has been applied for monitoring bacterial adhesion and biofilm formation, indirectly supporting the possibility that alterations in microbial biomass or biofilm architecture may contribute to changes in spectral characteristics (49). Interestingly, within the visible spectral range, hemoglobin absorption and local microvascular conditions can directly influence diffuse reflectance. Oxyhemoglobin exhibits characteristic absorption peaks around 542 and 577 nm in oral mucosal tissues, and variations in microvascular density and blood volume can significantly alter reflectance intensity in this spectral region (50). Therefore, the correlations observed between *Pseudoleptotrichia*, *Xylanibacter*, *Gemella* and spectral features in the 580~620 nm range may reflect microbiota-associated local inflammatory or oxygenation responses rather than direct effects of microbial components on optical signals. Importantly, chronic low-grade inflammation is a key characteristic of diabetes, and inflammatory alterations may affect tongue tissue properties, thereby modifying spectral absorption patterns (50, 51). However, due to limitations in the current experimental design, quantitative measurements of tongue coating thickness, surface moisture, and local microvascular perfusion were not performed. Therefore, it remains unclear whether the observed spectral variations primarily originate from physical alterations of the tongue coating, hemoglobin-related microcirculatory changes, tissue hydration status, microbial metabolic activity, or interactions among these factors. Accordingly, the microbiome-hyperspectral associations identified in this study should be interpreted primarily as correlated manifestations of alterations in the tongue microenvironment rather than evidence of direct causal effects of specific microorganisms on spectral reflectance. Future studies include larger cohorts and integrate quantitative assessments of tongue coating thickness and moisture, local microcirculation imaging, systemic and local inflammatory biomarkers, bacterial biomass, and tongue coating metabolomics are warranted to further elucidate the physiological mechanisms underlying these spectral features.

This study constructed T2DM disease status identification models based on hyperspectral tongue data combined with three machine learning algorithms: SVM, RF, and PLS-DA, achieving favorable identification performance. Different spectral preprocessing methods had different impact on model classification performance, with S-G smoothing achieving superior classification performance across multiple tongue regions. Hyperspectral data acquisition is susceptible to various sources of interference, including random noise, instrumental response variations, and light scattering effects, which may reduce the reliability of extracted spectral information. The S-G smoothing algorithm reduces high-frequency noise through local polynomial fitting while preserving the peak characteristics and spectral trends of the original curves. Therefore, S-G smoothing improves spectral signal stability. In addition, hyperspectral datasets typically contain numerous continuous wavelength variables, among which some variables may exhibit strong correlations or redundancy. Directly inputting all wavelengths into classification models may increase model complexity and introduce a risk of overfitting. Therefore, PLS-DA identifies latent variables associated with class information, reducing data dimensionality while retaining the major discriminative spectral features, thereby improving model generalization ability.

Notably, significant differences were observed in the discriminative ability among different tongue regions, with the tongue middle region achieving the best classification performance. Compared with the tongue tip and lateral regions, the tongue middle region is less affected by tongue movement and boundary structural variations, and it generally exhibits relatively thicker and more homogeneous tongue coating coverage, and its microenvironment may facilitate microbial colonization and metabolite accumulation, which could contribute to its greater sensitivity to metabolic alterations. Therefore, spectral features extracted from the tongue middle region may provide more stable and representative information. The optimal model further demonstrated discrimination capability for HC and T2DM samples, whereas its classification performance for IGR samples was relatively limited. IGR represents a transitional stage between normal glucose metabolism and diabetes, during which individual differences in pancreatic β-cell function, insulin sensitivity, and metabolic status may be substantial. Moreover, the number of IGR samples in the independent test set was limited (n = 3), which may have further increased uncertainty in model performance evaluation. Therefore, although the current model effectively distinguished healthy individuals from individuals with established T2DM, further validation with larger sample cohorts is required to improve the accurate identification of prediabetic states.

From a clinical perspective, the present findings suggest that hyperspectral tongue imaging combined with machine learning algorithms can extract potential disease-related information from spectral dimensions that are difficult to capture through conventional visual examination, providing a novel technical approach for screening T2DM patients. Compared with traditional blood glucose measurements, this approach offers advantages including non-invasiveness, rapid acquisition, and repeatability, and may serve as a potential screening or auxiliary assessment tool for individuals at high risk of diabetes. However, several limitations should be acknowledged. First, the sample size of this study was relatively limited, particularly for the IGR group. Although *post hoc* power analysis suggested that the observed alpha diversity differences represented relatively large effects, the small IGR subgroup may have limited the detection of subtle microbial alterations. Future studies could enhance result reliability by expanding the sample size. Second, the model was developed using single-center data. Subsequent research should extend to multi-center, multi-regional collaborative studies with external validation to strengthen the external validity of the conclusions. In addition, the biological mechanisms linking hyperspectral signals with tongue coating microbiota, tissue structures, and metabolic alterations remain to be further elucidated. Future studies integrating hyperspectral tongue imaging with microbiomics, metabolomics, and clinical parameters through multimodal analysis may further improve the accuracy of early diabetes risk prediction.

## 5 Conclusion

Based on hyperspectral imaging technology, this study compared tongue images among HC, IGR, and T2DM participants, revealing spectral curve differences in the wavelength range of 680~750 nm among the three groups. Concurrently, using 16S rRNA technology, characteristic differences in oral microbiota diversity and species composition were identified among the three populations, providing robust support for elucidating the pathogenesis of T2DM and its early diagnosis. Further correlation analysis of hyperspectral tongue data and tongue coating microbial community structure changes among the three groups revealed associations between key tongue coating genera and hyperspectral tongue features, providing a theoretical basis for the application of hyperspectral tongue image features in early screening of T2DM disease status. To explore the feasibility of T2DM disease status identification based on hyperspectral tongue images, this study constructed T2DM predictive models combining three machine learning algorithms: SVM, RF, and PLS-DA. Among the preprocessing, S-G smoothing improved spectral feature stability and classification performance. Following PLS-DA dimensionality reduction, the SVM model based on tongue middle region spectral features achieved the best overall performance, with an accuracy of 0.89655 and an AUC of 0.80290 on the independent test set, providing a novel noninvasive approach for auxiliary diagnosis of T2DM.

## Data Availability

The 16S rRNA sequencing data generated in this study have been deposited in the NCBI Sequence Read Archive (SRA) under BioProject accession number PRJNA1525526 and are publicly available at https://www.ncbi.nlm.nih.gov/bioproject/PRJNA1525526.
