# Correction: Correlation analysis between tongue hyperspectral imaging and tongue coating microbiota and feasibility study for early screening of type 2 diabetes mellitus status

**DOI:** 10.3389/fendo.2026.2030428

**Published:** 2026-10-30

**Authors:** Zhao-wei Liu, Yan Zhang, Lei Fu, Jing-ya Xie, Xin-yu Zhang, Ming Liu, Jing Zhao

**Affiliations:** 1 Tianjin University of Traditional Chinese Medicine, Tianjin, China; 2 Binhai New Area Hospital of TCM, Tianjin, China; 3 Institute of Biomedical Engineering, Chinese Academy of Medical Sciences and Peking Union Medical College, Tianjin, China

**Keywords:** disease risk prediction, hyperspectral tongue imaging, machine learning algorithms, tongue coating microbiota, type 2 diabetes mellitus

The funder “National Natural Science Foundation of China, Grant Nos. 81973699, 82274361” to “Jing Zhao” was erroneously omitted.

The correct **Funding** statement is: “This work was supported by the National Natural Science Foundation of China (Grant Nos. 81973699 and 82274361); the National Science and Technology Innovation 2030, Noncommunicable Chronic Diseases-National Science and Technology Major Project (Grant Nos. 2024ZD0524300 and 2024ZD0524301); and the project Exploration of a Traditional Chinese Medicine-Integrated Management Model for Elderly Patients with Chronic Comorbidities Based on Frailty Assessment (Project No. 2023200)”.

The original version of this article has been updated.

