# Supplementary material for: Correlation analysis between tongue hyperspectral imaging and tongue coating microbiota and feasibility study for early screening of type 2 diabetes mellitus status
Source: Front Endocrinol (Lausanne). 2026 Sep 18;17:1936566. doi: 10.3389/fendo.2026.1936566 (PMC13630686; doi:10.3389/fendo.2026.1936566)
Supplement: Supplementary file 1 [file Table1.docx]

**Supplementary Table 1** Multinomial logistic regression analysis of factors associated with disease status

| 变量 | IGR vs HC | | T2DM vs HC | |
| --- | --- | --- | --- | --- |
|  | OR (95%CI) | *P* | OR (95%CI) | *P* |
| Age | 1.073 (1.008 - 1.142) | 0.027 | 1.078 (1.025 - 1.134) | 0.004 |
| BMI | 1.085 (0.913 - 1.289) | 0.353 | 1.170 (1.022 – 1.340) | 0.023 |
| Sex (male) | 3.989 (0.696 – 22.864) | 0.120 | 12.667 (3.106 – 51.654) | 0.000 |
| Smoking (never) | 2.206 (0.289 – 16.830) | 0.445 | 0.537 (0.116 – 2.498) | 0.428 |
| Alcohol (never) | 0.468 (0.085 – 2.580) | 0.383 | 0.836 (0.209 – 3.338) | 0.800 |

Note: HC was used as the reference category. ORs were adjusted for age, BMI, sex, smoking status, and alcohol consumption.

**Supplementary Table 2** Effect of different preprocessing methods on model performance in the tongue middle region

| Classifier | Preprocessing | Accuracy | Recall | Specificity | Macro F1-Score | AUC |
| --- | --- | --- | --- | --- | --- | --- |
| SVM | NONE | 0.8063 | 0.8375 | 0.9056 | 0.8110 | 0.9287 |
|  | **S-G smoothing** | **0.8813** | **0.8833** | **0.9361** | **0.8796** | **0.9434** |
|  | SVN | 0.8000 | 0.8125 | 0.8986 | 0.7999 | 0.9168 |
|  | MSC | 0.6375 | 0.5792 | 0.7917 | 0.5928 | 0.7770 |
|  | 1^st^D | 0.8688 | 0.8708 | 0.9333 | 0.8619 | 0.9261 |
|  | S-G smoothing +SNV | 0.8625 | 0.8583 | 0.9250 | 0.8598 | 0.9337 |
|  | S-G smoothing +MSC | 0.6375 | 0.5625 | 0.7819 | 0.5791 | 0.7703 |
|  | S-G smoothing +1^st^D | 0.8313 | 0.8375 | 0.9111 | 0.8304 | 0.9153 |
| RF | NONE | 0.8188 | 0.8125 | 0.9014 | 0.8102 | 0.9198 |
|  | S-G smoothing | 0.8000 | 0.7708 | 0.8819 | 0.7807 | 0.9244 |
|  | SVN | 0.7875 | 0.7542 | 0.8806 | 0.7644 | 0.7879 |
|  | MSC | 0.6250 | 0.6375 | 0.8028 | 0.6279 | 0.7630 |
|  | 1^st^D | 0.7938 | 0.7875 | 0.8889 | 0.7853 | 0.9032 |
|  | S-G smoothing +SNV | 0.7875 | 0.7542 | 0.8750 | 0.7684 | 0.9011 |
|  | S-G smoothing +MSC | 0.6625 | 0.6458 | 0.8153 | 0.6504 | 0.7934 |
|  | S-G smoothing +1^st^D | 0.7813 | 0.7792 | 0.8806 | 0.7770 | 0.9064 |
| PLS-DA | NONE | 0.7375 | 0.7708 | 0.8722 | 0.7386 | 0.8904 |
|  | S-G smoothing | 0.7750 | 0.7708 | 0.8847 | 0.7625 | 0.9046 |
|  | SVN | 0.7250 | 0.7500 | 0.8653 | 0.7260 | 0.8909 |
|  | MSC | 0.4938 | 0.4917 | 0.7250 | 0.4865 | 0.6128 |
|  | 1^st^D | 0.7625 | 0.7917 | 0.8861 | 0.7635 | 0.8804 |
|  | S-G smoothing +SNV | 0.7813 | 0.7833 | 0.8847 | 0.7776 | 0.8992 |
|  | S-G smoothing +MSC | 0.4500 | 0.4375 | 0.7014 | 0.4303 | 0.5866 |
|  | S-G smoothing +1^st^D | 0.7375 | 0.7625 | 0.8736 | 0.7365 | 0.8796 |

**Supplementary Table 3** Effect of different preprocessing methods on model performance in the tongue tip region

| Classifier | Preprocessing | Accuracy | Recall | Specificity | Macro F1-Score | AUC |
| --- | --- | --- | --- | --- | --- | --- |
| SVM | NONE | 0.7188 | 0.7250 | 0.8556 | 0.7028 | 0.8604 |
|  | **S-G smoothing** | **0.8063** | **0.8000** | **0.8931** | **0.7996** | **0.8931** |
|  | SVN | 0.7250 | 0.7292 | 0.8542 | 0.7167 | 0.8665 |
|  | MSC | 0.6625 | 0.6458 | 0.8125 | 0.6495 | 0.7647 |
|  | 1^st^D | 0.7313 | 0.7375 | 0.8611 | 0.7279 | 0.8464 |
|  | S-G smoothing +SNV | 0.7688 | 0.7625 | 0.8722 | 0.7578 | 0.8736 |
|  | S-G smoothing +MSC | 0.6188 | 0.5875 | 0.7833 | 0.5842 | 0.7252 |
|  | S-G smoothing +1^st^D | 0.7250 | 0.7292 | 0.8514 | 0.7158 | 0.8835 |
| RF | NONE | 0.7500 | 0.7167 | 0.8556 | 0.7267 | 0.8814 |
|  | S-G smoothing | 0.7375 | 0.7000 | 0.8472 | 0.7109 | 0.8610 |
|  | SVN | 0.6938 | 0.6667 | 0.8347 | 0.6556 | 0.8298 |
|  | MSC | 0.6375 | 0.6417 | 0.8083 | 0.6340 | 0.7677 |
|  | 1^st^D | 0.6813 | 0.6583 | 0.8250 | 0.6553 | 0.8263 |
|  | S-G smoothing +SNV | 0.6750 | 0.6542 | 0.8264 | 0.6478 | 0.8081 |
|  | S-G smoothing +MSC | 0.5938 | 0.5625 | 0.7736 | 0.5688 | 0.7273 |
|  | S-G smoothing +1^st^D | 0.7313 | 0.7208 | 0.8500 | 0.7187 | 0.8720 |
| PLS-DA | NONE | 0.6500 | 0.6833 | 0.8278 | 0.6454 | 0.8072 |
|  | S-G smoothing | 0.6813 | 0.7083 | 0.8444 | 0.6727 | 0.8007 |
|  | SVN | 0.6625 | 0.6667 | 0.8264 | 0.6432 | 0.7793 |
|  | MSC | 0.5813 | 0.6083 | 0.7903 | 0.5745 | 0.7069 |
|  | 1^st^D | 0.6813 | 0.7042 | 0.8417 | 0.6751 | 0.7978 |
|  | S-G smoothing +SNV | 0.6688 | 0.7000 | 0.8347 | 0.6642 | 0.7812 |
|  | S-G smoothing +MSC | 0.5563 | 0.5417 | 0.7639 | 0.5315 | 0.6399 |
|  | S-G smoothing +1^st^D | 0.6438 | 0.6750 | 0.8208 | 0.6352 | 0.8090 |

**Supplementary Table 4** Effect of different preprocessing methods on model performance in the tongue left region

| Classifier | Preprocessing | Accuracy | Recall | Specificity | Macro F1-Score | AUC |
| --- | --- | --- | --- | --- | --- | --- |
| SVM | NONE | 0.7938 | 0.8000 | 0.8917 | 0.7887 | 0.9059 |
|  | **S-G smoothing** | **0.8375** | **0.8292** | **0.9097** | **0.8293** | **0.9092** |
|  | SVN | 0.7188 | 0.7333 | 0.8556 | 0.7132 | 0.8781 |
|  | MSC | 0.5688 | 0.5000 | 0.7431 | 0.4998 | 0.7069 |
|  | 1^st^D | 0.7375 | 0.7417 | 0.8597 | 0.7320 | 0.8497 |
|  | S-G smoothing +SNV | 0.7625 | 0.7542 | 0.8681 | 0.7522 | 0.8937 |
|  | S-G smoothing +MSC | 0.6125 | 0.5375 | 0.7625 | 0.5518 | 0.7230 |
|  | S-G smoothing +1^st^D | 0.7313 | 0.7292 | 0.8542 | 0.7221 | 0.8390 |
| RF | NONE | 0.7313 | 0.7125 | 0.8514 | 0.7106 | 0.8604 |
|  | S-G smoothing | 0.7813 | 0.7542 | 0.8722 | 0.7670 | 0.8549 |
|  | SVN | 0.7250 | 0.7167 | 0.8458 | 0.7174 | 0.8523 |
|  | MSC | 0.6000 | 0.5792 | 0.7861 | 0.5773 | 0.7304 |
|  | 1^st^D | 0.6813 | 0.6750 | 0.8278 | 0.6677 | 0.8129 |
|  | S-G smoothing +SNV | 0.7625 | 0.7292 | 0.8611 | 0.7395 | 0.8605 |
|  | S-G smoothing +MSC | 0.6188 | 0.5917 | 0.7861 | 0.5963 | 0.7529 |
|  | S-G smoothing +1^st^D | 0.6938 | 0.7000 | 0.8361 | 0.6845 | 0.8630 |
| PLS-DA | NONE | 0.7000 | 0.7333 | 0.8514 | 0.6985 | 0.8271 |
|  | S-G smoothing | 0.7000 | 0.7125 | 0.8458 | 0.6933 | 0.8364 |
|  | SVN | 0.7188 | 0.7292 | 0.8556 | 0.7120 | 0.8471 |
|  | MSC | 0.5375 | 0.4667 | 0.7319 | 0.4620 | 0.6079 |
|  | 1^st^D | 0.6438 | 0.6792 | 0.8222 | 0.6468 | 0.7684 |
|  | S-G smoothing +SNV | 0.7125 | 0.7125 | 0.8472 | 0.6994 | 0.8436 |
|  | S-G smoothing +MSC | 0.5500 | 0.4542 | 0.7292 | 0.4437 | 0.6360 |
|  | S-G smoothing +1^st^D | 0.6375 | 0.6792 | 0.8181 | 0.6381 | 0.7798 |

**Supplementary Table 5** Effect of different preprocessing methods on model performance in the tongue rigth region

| Classifier | Preprocessing | Accuracy | Recall | Specificity | Macro F1-Score | AUC |
| --- | --- | --- | --- | --- | --- | --- |
| SVM | NONE | 0.7125 | 0.7292 | 0.8514 | 0.7042 | 0.8397 |
|  | **S-G smoothing** | **0.7875** | **0.7875** | **0.8875** | **0.7800** | **0.8978** |
|  | SVN | 0.7438 | 0.7458 | 0.8639 | 0.7330 | 0.8431 |
|  | MSC | 0.7125 | 0.6667 | 0.8292 | 0.6767 | 0.8431 |
|  | 1^st^D | 0.7188 | 0.7333 | 0.8569 | 0.7140 | 0.8486 |
|  | S-G smoothing +SNV | 0.7688 | 0.7583 | 0.8750 | 0.7575 | 0.9001 |
|  | S-G smoothing +MSC | 0.7313 | 0.6667 | 0.8292 | 0.6715 | 0.8385 |
|  | S-G smoothing +1^st^D | 0.7563 | 0.7583 | 0.8708 | 0.7436 | 0.8863 |
| RF | NONE | 0.7313 | 0.6875 | 0.8403 | 0.6977 | 0.8513 |
|  | S-G smoothing | 0.7750 | 0.7375 | 0.8681 | 0.7425 | 0.9050 |
|  | SVN | 0.7438 | 0.7208 | 0.8583 | 0.7169 | 0.8377 |
|  | MSC | 0.6375 | 0.6167 | 0.8014 | 0.6133 | 0.8181 |
|  | 1^st^D | 0.6313 | 0.6208 | 0.8056 | 0.6082 | 0.8013 |
|  | S-G smoothing +SNV | 0.7563 | 0.7167 | 0.8639 | 0.7236 | 0.8790 |
|  | S-G smoothing +MSC | 0.6938 | 0.6625 | 0.8208 | 0.6695 | 0.8422 |
|  | S-G smoothing +1^st^D | 0.7375 | 0.7083 | 0.8514 | 0.7027 | 0.8828 |
| PLS-DA | NONE | 0.6438 | 0.6625 | 0.8167 | 0.6352 | 0.8043 |
|  | S-G smoothing | 0.7125 | 0.7292 | 0.8528 | 0.7044 | 0.8492 |
|  | SVN | 0.6688 | 0.6792 | 0.8250 | 0.6536 | 0.7814 |
|  | MSC | 0.5563 | 0.5625 | 0.7722 | 0.5422 | 0.6648 |
|  | 1^st^D | 0.6563 | 0.6750 | 0.8264 | 0.6492 | 0.8235 |
|  | S-G smoothing +SNV | 0.7000 | 0.7083 | 0.8431 | 0.6930 | 0.8657 |
|  | S-G smoothing +MSC | 0.5125 | 0.4875 | 0.7361 | 0.4789 | 0.6625 |
|  | S-G smoothing +1^st^D | 0.6563 | 0.6542 | 0.8208 | 0.6355 | 0.8418 |

**Supplementary Table 6**Effect of different preprocessing methods on model performance in the tongue surface region

| Classifier | Preprocessing | Accuracy | Recall | Specificity | Macro F1-Score | AUC |
| --- | --- | --- | --- | --- | --- | --- |
| SVM | NONE | 0.7750 | 0.7875 | 0.8861 | 0.7687 | 0.8983 |
|  | **S-G smoothing** | **0.8313** | **0.8333** | **0.9097** | **0.8279** | **0.9175** |
|  | SVN | 0.7250 | 0.7333 | 0.8569 | 0.7148 | 0.8490 |
|  | MSC | 0.6313 | 0.5917 | 0.7833 | 0.5990 | 0.7363 |
|  | 1^st^D | 0.7500 | 0.7750 | 0.8778 | 0.7514 | 0.8723 |
|  | S-G smoothing +SNV | 0.7813 | 0.7792 | 0.8819 | 0.7755 | 0.8739 |
|  | S-G smoothing +MSC | 0.5750 | 0.5042 | 0.7417 | 0.5136 | 0.7383 |
|  | S-G smoothing +1^st^D | 0.8125 | 0.8083 | 0.9028 | 0.8025 | 0.9154 |
| RF | NONE | 0.7875 | 0.7792 | 0.8861 | 0.7759 | 0.9257 |
|  | S-G smoothing | 0.7938 | 0.7750 | 0.8847 | 0.7805 | 0.9181 |
|  | SVN | 0.7875 | 0.7708 | 0.8806 | 0.7720 | 0.8929 |
|  | MSC | 0.5813 | 0.5875 | 0.7736 | 0.5780 | 0.7441 |
|  | 1^st^D | 0.7750 | 0.7583 | 0.8736 | 0.7661 | 0.8872 |
|  | S-G smoothing +SNV | 0.7375 | 0.7208 | 0.8611 | 0.7138 | 0.8694 |
|  | S-G smoothing +MSC | 0.6313 | 0.6083 | 0.7903 | 0.6116 | 0.7730 |
|  | S-G smoothing +1^st^D | 0.7813 | 0.7542 | 0.8764 | 0.7606 | 0.9136 |
| PLS-DA | NONE | 0.6875 | 0.7292 | 0.8486 | 0.6865 | 0.8549 |
|  | S-G smoothing | 0.7938 | 0.8000 | 0.8944 | 0.7855 | 0.9030 |
|  | SVN | 0.7125 | 0.7250 | 0.8542 | 0.7041 | 0.8483 |
|  | MSC | 0.4688 | 0.4625 | 0.7167 | 0.4553 | 0.5833 |
|  | 1^st^D | 0.6750 | 0.7083 | 0.8417 | 0.6719 | 0.8529 |
|  | S-G smoothing +SNV | 0.7688 | 0.7625 | 0.8833 | 0.7514 | 0.8968 |
|  | S-G smoothing +MSC | 0.4375 | 0.3875 | 0.6819 | 0.3786 | 0.5872 |
|  | S-G smoothing +1^st^D | 0.7500 | 0.7667 | 0.8764 | 0.7433 | 0.8934 |
